# Can Contrast-Enhanced Spectral Mammography (CESM) Reduce Benign Breast Biopsy?

**DOI:** 10.1155/2022/7087408

**Published:** 2022-03-24

**Authors:** Amanda Ling Fung Liew, Hollie Mei Yeen Lim, Elizabeth Chun Mei Fok, Siu Cheng Loke, Ern Yu Tan, Bee Kiang Chong, Yeong Shyan Lee, Patrick Mun Yew Chan, Niketa Chotai

**Affiliations:** ^1^National Cancer Centre Singapore, 11 Hospital Cres, Singapore; ^2^Tan Tock Seng Hospital, 11 Jln Tan Tock Seng, Singapore; ^3^Lee Kong Chian School of Medicine, Nanyang Technological University, 11 Mandalay Road, Singapore; ^4^Institute of Molecular and Cell Biology, Agency for Science, Technology and Research (A^∗^STAR), 61 Biopolis Dr, Singapore; ^5^RadLink Diagnostic Imaging Singapore, 08-08, 290 Orchard Road, Singapore

## Abstract

**Objectives:**

To evaluate the potential of contrast-enhanced spectral mammography (CESM) in reducing benign breast biopsy rate, thereby improving resource utilization. To explore its potential as a value-adding modality in the management of BI-RADS 4/5 lesions.

**Materials and Methods:**

This was a prospective study conducted between July 2016 and September 2018. Patients with BI-RADS 4/5 lesions detected on conventional imaging (mammogram, digital breast tomosynthesis, and ultrasound) were enrolled for adjunct CESM. Histopathologic correlation was done for all lesions. Additional suspicious lesions detected on CESM were all identified on second-look ultrasound and subsequently biopsied. Images were evaluated independently by two radiologists trained in breast imaging using BI-RADS classification. Presence of enhancement on CESM, BI-RADS score, and histopathology of each lesion were analyzed and tested with the chi-square/fisher-exact test for statistical significance.

**Results:**

The study included 105 lesions in 63 participants—1 man and 62 women, an average age of 53.7 ± 10.8 years. On CESM, 22 (20.9%) of the lesions did not show enhancement. All 22 lesions had been classified as BI-RADS 4A and were subsequently proven to be benign. Of the remaining 83 enhancing lesions, 54 (65.1%) were malignant and 29 (34.9%) were benign (*p* < 0.05). CESM detected 6 additional lesions which were not identified on initial conventional imaging. Four of these were proven malignant and were in a different quadrant than the primary lesion investigated.

**Conclusion:**

There is evidence that the absence of enhancement in CESM strongly favors benignity. It may provide the reporting radiologist with greater confidence in imaging assessment, especially in BI-RADS 4A cases, where a proportion of them are in actuality BI-RADS 3. Greater accuracy of BI-RADS grading can reduce nearly half of benign biopsies and allow better resource allocation. CESM also increases the detection rate of potentially malignant lesions, thereby changing the treatment strategies.

## 1. Introduction

As per the American College of Radiology Breast Imaging Reporting and Data System (ACR BI-RADS) guidelines, it is widely accepted that BI-RADS 4 and 5 lesions require tissue diagnosis. The likelihood of malignancy associated with BI-RADS 4 lesions, however, varies widely between 2% and 10% for BI-RADS 4A and between 50% and 95% for BI-RADS 4C lesions [1]. Currently, as the overall malignancy rate is considered high in all subgroups of the BI-RADS 4 category, all the lesions labeled as BI-RADS 4 need to undergo biopsy to exclude malignancy. Consequently, to ensure the safety of the entire group, one needs to accept nearly 65% of benign biopsy results. Also, lesions with low suspicion of malignancy, i.e., BI-RADS 4A, may delay the biopsy of more suspicious lesions, i.e., BI-RADS 4C, due to resource division.

The overall annual utilization rate of breast biopsies is reported to be 62.6 per 10,000 women per year, with the age-adjusted incidence of benign breast biopsies at 38.9 per 10,000 women, equating to 62.1% of total biopsies [2]. Our center experienced a similar situation where approximately 65% of the total ultrasound-guided breast biopsies performed in a year, yielded benign histology.

A benign biopsy renders physical, financial, and psychological stress to the patient and increases healthcare burden [2–4]. More than one-third of older women continue to report at least one negative psychological consequence of undergoing a benign breast biopsy 6 months later, and 44% report anxiety about future mammograms [2].

In recent years, contrast-enhanced spectral mammography (CESM) has emerged as a promising imaging technique. There is evidence that CESM, apart from having the conventional mammographic utility in detecting lesions and calcifications, is also able to provide functional information with regard to lesional enhancement by leveraging on the concept of tumoural neoangiogenesis, similar in that sense to breast magnetic resonance imaging (MRI) [5–7]. Several studies have demonstrated the superior sensitivity and low false positive rates of CESM and have also explored its potential as an alternative to MRI breast imaging in breast cancer staging and problem solving [5–9]. Limited local data is available regarding its value in clinical use and its potential in reducing benign biopsy rates.

Our study aims to evaluate the usefulness of CESM in reducing the benign biopsy rate, thereby enabling better allocation of resources. We also explore the potential of CESM as a value-adding modality in the clinical management of BI-RADS 4 and 5 lesions.

## 2. Materials and Methods

This prospective study was approved by our local Institutional Review Board (Ethics Committee- DSRB reference number 2016/01388). All study participants included in the study provided written informed consent at the time of enrollment.

The study was carried out from July 2016 to September 2018 in a tertiary breast unit in a diagnostic setting.

Patients diagnosed with BI-RADS 4 and 5 lesions on conventional imaging (full-field digital mammography/digital breast tomosynthesis (DBT) and ultrasound) were identified. The exclusion criteria were patients who were younger than 30 years of age, pregnant or lactating, patients with three or more drug allergies, active asthma, or compromised renal function (eGFR < 30). Lesions with mammographic calcifications only were excluded. Eligible participants who were willing to participate were consented, enrolled, and referred for CESM and ultrasound-guided biopsy.

CESM was performed on the same day for most patients, or within one week prior to the scheduled image-guided biopsy. Regardless of the findings on CESM, ultrasound-guided biopsy was carried out for all lesions. The addition of CESM resulted in the detection of a few additional indeterminate enhancing lesions. These additional lesions were all identified on second-look ultrasound and subsequently biopsied under ultrasound guidance.

**Figure 1:**
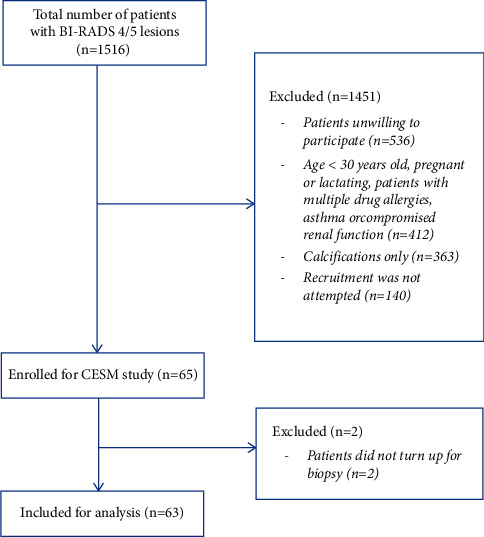
Flowchart of number of participants included.

The histopathology results were retrieved from the patient's electronic record. The biopsy result was reviewed by the assessing breast radiologists and/or breast surgeons. As per our local hospital's practice, papillary lesions, flat epithelial atypia (FEA), and atypical ductal hyperplasia (ADH) on biopsy were surgically excised. The histological analysis of the lesions was based on biopsy results or final histology results. Lesions without histopathological correlation were excluded from the final analysis (Figure 1).

### 2.1. Technical Aspects of CESM Image Acquisition

Examinations were acquired using a full-field digital mammography system, Senographe Essential (version 56.21.3) (GE Healthcare, Buc, France, 2016) with CESM capability. 1.5 ml/kg of Omnipaque 350 (350 mg iodine/ml) was injected at a rate of 3 ml/second through a power injector. After 2 minutes, images were acquired with a mediolateral oblique (MLO) view of the normal breast, followed by a MLO view of the abnormal breast, a craniocaudal (CC) view of the abnormal breast, and a CC view of the normal breast. Equipped with the knowledge that rapid enhancement is associated with higher probability of malignancy [10, 11], we placed the lesion of concern in the middle of the sequence in a bid to capture the peak of its enhancement, if present. CESM being a dual-energy technique, two exposures were acquired for each view, one with low (26–31 kVp) and one with high (45–49 kVp) energy. After acquisition, a specific weighted recombination of low- and high-energy images was recombined into “iodine” images with subtraction of glandular tissue to improve lesion uptake visualization [20]. All 4 images were taken within 6–7 minutes.

### 2.2. Analysis

**Figure 2:**
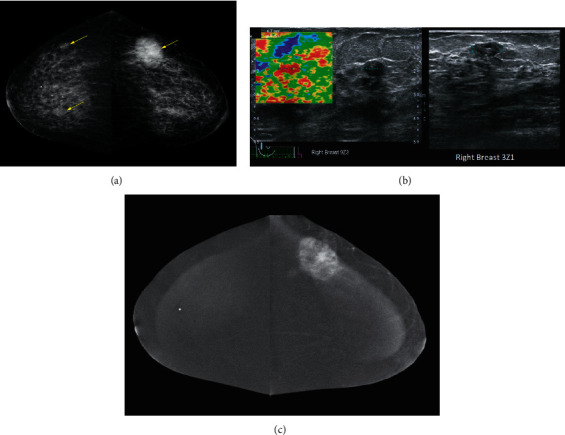
A 60-year-old woman with a palpable left breast lump. (a) Craniocaudal views of both breasts showed masses in the outer and inner halves of the right breast and a large mass associated with suspicious calcifications in the outer half of the left breast (yellow arrows). The left breast mass had yielded invasive ductal carcinoma on biopsy. (b) Selected US images of the right breast showed a stiff, solid nodule in the 0900 position of the right breast and another hypoechoic nodule with a cystic component with a mildly angular margin in the 0300 position of the right breast, corresponding to the masses seen on mammogram. In the context of biopsy proven contralateral cancer, they were initially categorized as BI-RADS 4A. (c) Subtracted CESM image in the craniocaudal view showed that both lesions in the right breast did not enhance. Note the left breast cancer showed intense enhancement. US guided biopsy of both lesions in the right breast yielded fibroadenomas. With the absence of enhancement, both of the lesions in the right breast, which were initially categorized as BI-RADS 4A, could have been downgraded to BI-RADS 3. US = ultrasound, BI-RADS = breast imaging reporting and data system and CESM = contrast-enhanced spectral mammography.

**Figure 3:**
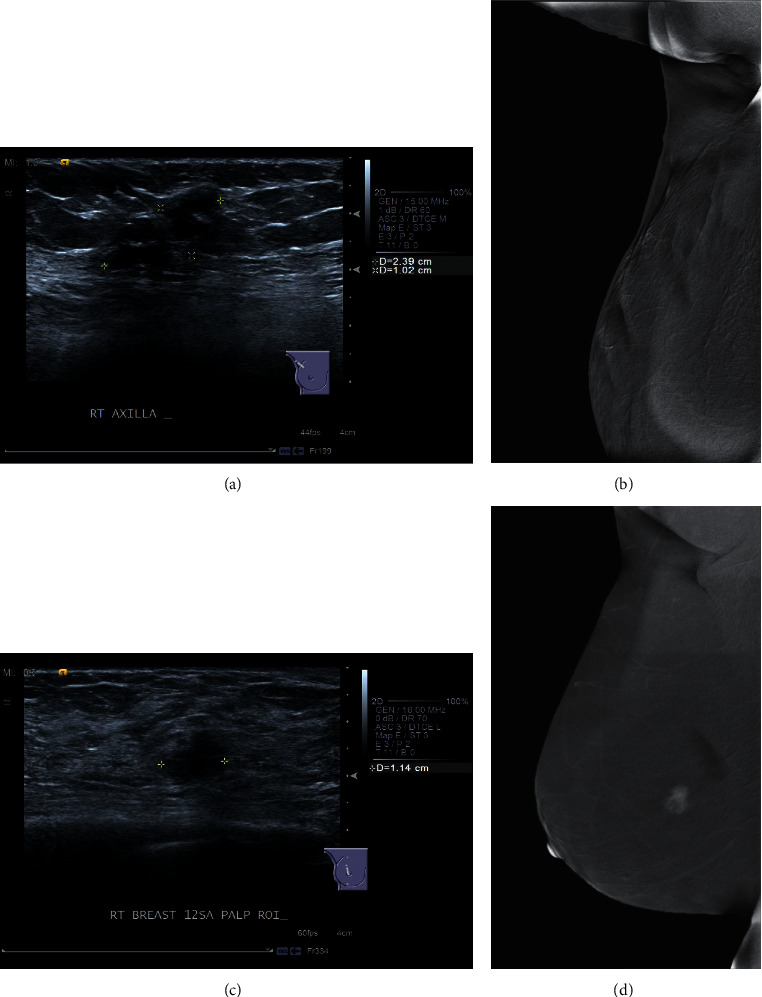
Example of 2 cases where the addition of CESM had helped in the clinical management of contentious BI-RADS 4 lesions. Both patients had prior wide local excision for malignancy and presented with a new palpable lump in the ipsilateral breast. (a) Selected US image in the first participant showed a new, lobulated hypoechoic nodule with cystic component. (b) It did not enhance on CESM and US-guided biopsy yielded benign histology. Subsequent follow-ups after 4 years showed a stable lesion. (c) Selected US image in another participant showed a new, irregular, nonparallel, hypoechoic nodule with angular margins. (d) It showed moderate to intense enhancement on CESM. A free-hand biopsy previously performed by the surgeon had yielded a benign result. Imaging features and the presence of moderate to intense enhancement on CESM had enabled the radiologist to confidently deem the result to be discordant. A repeat biopsy was performed under US guidance, and the lesion was indeed proven to be malignant. US = ultrasound and CESM = contrast-enhanced spectral mammography.

All study participants had mammogram, ultrasound, and CESM studies. All mammographic, ultrasound, and CESM images were evaluated independently by two radiologists with breast imaging training, both blinded to the final histopathology result. The first reader has more than 10 years of experience in breast radiology with 5 years of experience in CESM, while the second reader has 4 years of experience in breast radiology and received training in CESM prior to the commencement of the study. A single consensus was obtained in cases of disagreement. The BI-RADS score before and after the addition of CESM was recorded for each lesion. For the assessment of CESM, the same criteria for morphology were used as described in the BI-RADS lexicon for mammography (low-energy images). The presence or absence of enhancement was assessed on the subtracted images, and the intensity of enhancement was assessed visually and categorized as mild to moderate to intense. Examples showing the moderate and intense enhancement are shown in Figures2(c) and 3(d).

### 2.3. Statistical Analysis

The relationship between the degree of enhancement in CESM, the BI-RADS score before and after CESM, and the histopathology of each lesion was analyzed. A true positive result was concluded when the presence of enhancement was matched with malignant pathology; a true negative result was concluded when the absence of enhancement was matched with benign pathology. A false positive result was concluded when the presence of enhancement was matched with benign pathology, while a false negative result was concluded when the absence of enhancement was matched with malignant pathology. The sensitivity, specificity, negative predictive value, positive predictive value, and accuracy of CESM in the detection of breast cancer were analyzed.

The relationship between the enhancements in CESM with the final nature of the lesion was tested using the chi-square test. If one of the cells in the 2 × 2 contingency table had a value of 0, the Fisher exact test was used instead. *p* values of <0.05 were used to determine the statistical significance of both tests (IBM SPSS Statistics, Version 22.0, 2013).

## 3. Results

105 lesions in 63 participants—1 man and 62 women—were included. The median age was 53.7 (a range of 33–81 years). 44 participants (69.8%) were Chinese, 8 (12.7%) were Malay, 7 (11.1%) were Indian, and 4 (6.3%) were of other ethnicities. 51 participants (81.0%) were symptomatic with 40 (63.5%) presenting with palpable lump, 6 (9.5%) with mastalgia, 3 (4.8%) with nipple retraction, and 2 (3.2%) with skin irritation. 12 (19.0%) participants were asymptomatic—7 (11.1%) had a new breast nodule or change in preexisting nodule on screening study, and 5 (7.9%) had incidental breast nodule detected on computed tomography scan for other clinical indications. Breast density BI-RADS classifications were A, B, C, and D for 1 (1.6%), 10 (15.9%), 42 (66.7%), and 10 (15.9%) patients, respectively.

On mammogram, 44 (41.9%) lesions were seen as a mass, 15 (14.3%) were mass with calcifications, 8 (7.6%) were mass with architectural distortion, and 38 (36.2%) lesions were mammographically occult. All the included lesions were visualized on ultrasound as masses.

Before the addition of CESM, 18 (17.1%) lesions were classified as BI-RADS 4A, 40 (38.1%) as BI-RADS 4B, 29 (27.6%) as BI-RADS 4C, and 12 (11.4%) as BI-RADS 5. 6 (5.7%) lesions were missed on the initial assessment.

**Figure 4:**
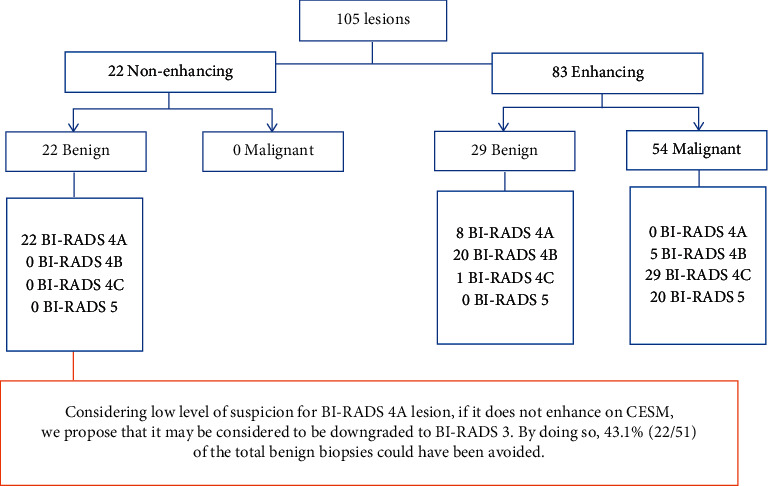
Enhancement in CESM and final nature of the lesions. BI-RADS = breast imaging reporting and data system and CESM = contrast-enhanced spectral mammography.

Combining the mammogram, ultrasound, and CESM findings, 30 (28.6%) lesions were classified as BI-RADS 4A, 25 (23.8%) as BI-RADS 4B, 30 (28.6%) as BI-RADS 4C, and 20 (19.0%) as BI-RADS 5. Of the 105 lesions, 22 (21.0%) did not enhance, all of which were BI-RADS 4A and were subsequently found to be benign (Figure 4). None of these were palpable.

Of the remaining 83 enhancing lesions, 54 (65.1%) were malignant and 29 (34.9%) were benign (Figure 4 and Table 1). 10 lesions (8 benign and 2 malignant) showed mild enhancement. 73 lesions (21 benign and 52 malignant) demonstrated moderate to intense enhancement (*p* value = 0.001). 51 out of 54 malignant lesions were invasive tumour with a mean histologic size of 31 mm (range 10–80 mm); 9 were of grade 1, 24 were of grade 2, and 18 were of grade 3. The other 3 malignant lesions were ductal carcinoma in situ (DCIS) with a mean histology size of 11 mm (range 3–20 mm); 1 was of intermediate grade and 2 were of high grade.

Amongst the 29 false positive findings, 13 (44.8%) were fibroadenomas, 6 (20.7%) were intraductal papillomas, 4 (13.8%) were nodular adenosis, 2 (6.9%) were benign breast tissue, 1 (3.4%) was fat necrosis, 1 (3.4%) was flat epithelial atypia, 1 (3.4%) was benign phyllodes tumour, and 1 (3.4%) was ruptured cyst (Table 1). One BI-RADS 4C lesion was biopsied under ultrasound guidance and yielded fibroadenoma (considered discordant). The patient declined excisional biopsy and opted for continued follow-up. The lesion was stable on yearly surveillance for 3 years after detection. Of the 8 benign lesions that showed mild enhancement, 2 showed faint enhancement only in the later phase of CESM acquisition.

The diagnostic performance based on the criteria of enhancement alone is as shown in Table 2.  Figure 2 demonstrates examples of BI-RADS 4A lesions that could have been downgraded to BI-RADS 3.

CESM detected 6 additional indeterminate enhancing lesions which were not initially identified on the conventional assessment. They were all identified on second-look ultrasound and biopsied. Of the 6, 4 were proven malignant and located in a different quadrant from the primary lesion that was initially investigated. The other 2 were fibroadenomas.

With the addition of CESM, 16 lesions were assigned a higher BI-RADS score—1 from BI-RADS 4A to 4B, 7 from BI-RADS 4B to 4C, and 8 from BI-RADS 4C to 5. Of the 16 lesions, 7 showed moderate enhancement and 9 showed intense enhancement. 12 were invasive ductal carcinomas (IDCs), 2 were invasive lobular carcinomas (ILCs), 1 was nodular adenosis, and 1 was FEA.

With the addition of CESM, 15 lesions were assigned a lower BI-RADS score after CESM-13 from BI-RADS 4B to BI-RADS 4A and 2 from BI-RADS 4C to 4B. 8 lesions did not enhance, 4 lesions showed mild enhancement, and 3 lesions showed moderate enhancement. All 15 lesions were benign—3 fibroadenomas, 3 papillomas, 2 fat necrosis, 1 fibrocystic change, 1 sclerosing adenosis, 1 nodular adenosis, 1 radial scar, 1 ruptured cyst, 1 stromal fibrosis, and 1 benign breast tissue.

Addition of CESM helped in the clinical management of contentious BI-RADS 4 lesions in 2 participants (Figure 3). Both had a personal history of malignancy, prior wide local excision, and presented with a new palpable lump in the previously treated breast. The lesion that did not enhance on CESM (Figures 3(a) and 3(b)) yielded benign histology after ultrasound-guided biopsy. The absence of enhancement increased the radiologist's and clinician's confidence in the validation of the biopsy result. The patient opted for follow-up over a repeat biopsy or surgical excision. Follow-up after 4 years showed stability of the lesion. The lesion in the other participant showed moderate to intense enhancement on CESM (Figure 3(c) and 3(d)). It also yielded a benign result after free-hand biopsy by the surgeon. However, the presence of moderate to intense enhancement, together with its imaging features, significantly increased the radiologist's confidence in deciding that the biopsy result was discordant. A repeat biopsy under ultrasound guidance subsequently confirmed a malignant diagnosis.

## 4. Discussion

Lesions that did not enhance in our study were all non-palpable, BI-RADS 4A, and benign. Our data confirms that the absence of enhancement on CESM strongly supports the benignity of the lesion. Tagliafico et al. [12] reported a pooled sensitivity of 98% for CESM in detecting breast cancer in his meta-analysis, which included more than 900 lesions. Four out of the 8 included studies demonstrated a sensitivity of 100%, which is the same as our study [12–17]. Similar results with a sensitivity range of 83–100% for CESM were reported in the recent meta-analysis by Dromain et al. [18]. The high negative predictive value of CESM suggests that the absence of enhancement in CESM almost always excludes breast malignancy [18]. We propose that lesion that is both non-palpable and demonstrates low suspicion for malignancy morphologically (BI-RADS 4A) should be considered for a downgrade to BI-RADS 3 if there is absence of enhancement on CESM. The implementation of this would have avoided 22 out of 51 (43.1%) benign biopsies in our study.

Although not observed in our study, we acknowledge that there is a small percentage (2.0 to 5.5%) of false negative findings reported in the literature [12, 18–21]. A lesion which is out of the field of view of CESM is a recognized reason for a false negative finding [18, 20]. Ductal carcinoma in situ (DCIS), invasive lobular carcinomas (ILCs), and mucinous carcinomas showing only subtle or no enhancement have also been reported [12, 18–21].

In our institution, ultrasound-guided biopsy of a breast lesion costs about SGD 1000, whereas a CESM examination costs about SGD 100. There is an average of 600 ultrasound-guided biopsy procedures performed per year, out of which approximately 65% turn out to be benign, i.e., about (0.65 × 600) 390 biopsies. By adding CESM, we may hypothetically be able to avoid 43.1% of total benign biopsyies in our institution; absolute number of 168 (0.431 × 390). This translates to a total savings of approximately SGD151 200 (SGD 168,000 minus SGD 16,800) per year. These biopsy slots would be better allocated to patients with a higher suspicion of malignancy, thereby guiding treatment more effectively.

Though the incidence of clinical complications after breast biopsy is generally low, reported complications include haematoma, infection, vasovagal syncope, and pneumothorax [22]. It is also known that breast biopsy procedures are stressful for women. A benign biopsy may also increase a woman's anxiety for the next mammogram [1–3]. According to a survey by Lindfors et al., the self-reported stress level experienced by women followed up with imaging (i.e., BI-RADS 3) was significantly lower compared to women who underwent core biopsies [23]. CESM, by virtue of reducing benign biopsy rate, has the potential to reduce the unnecessary negative psychological impact towards patients.

CESM is able to identify multifocal malignancies with several studies demonstrating its sensitivity approaching that of MRI breast and with superior specificity [21, 27]. Lee-Felker et al. reported similar sensitivity between CESM and MRI (94% vs 99%, respectively) for detection of index lesions and higher sensitivity for CESM compared to MRI (100% vs 91%, respectively) for detection of secondary cancers [24]. The addition of CESM has resulted in more accurate local staging in our study when compared to conventional imaging. Six additional lesions were detected after CESM, of which 4 were proven malignant and were in a different quadrant than the primary lesion that was initially investigated. This resulted in a clinical upstage from single/multifocal disease to multicentric disease, thereby changing the surgical approach and treatment strategies [25, 26].

The addition of CESM may help to improve the accuracy and diagnostic confidence of BI-RADS subcategorization [12–16]. In our study, 14 out of 16 lesions (87.5%) that were upgraded to a higher score after CESM were subsequently found to be malignant, whereas all 15 lesions (100%) that were downgraded to a lower score after CESM were later found to be benign.

The addition of CESM also helped in the clinical management of BI-RADS 4 lesions by increasing the radiologist's confidence in suggesting the next appropriate management option when the histological result of the lesion was deemed discordant (Figure 3). The absence of enhancement in one lesion helped to support a benign biopsy result with the benignity of the lesion confirmed at further follow-up, whereas the presence of enhancement in the other lesion helped to refute a benign biopsy result with malignancy proven on repeat biopsy.

To date, the BI-RADS lexicon for MRI has been applied to CESM [5]. Apart from the shape, margin, and internal enhancement characteristics, we studied the relationship of intensity of enhancement on CESM with the final nature of a lesion. Our data suggest that malignant lesions show moderate to intense enhancement more frequently than benign lesions. In addition, we postulate that the pattern of enhancement may be inferred by analyzing the images according to the sequence of image acquisition. There were 2 benign lesions which showed faint enhancement only in the later phase of CESM acquisition. This may suggest progressive enhancement (i.e., type I equivalent in MRI enhancement kinetic curve) rather than the more worrisome rapid contrast uptake with wash out (i.e., type III equivalent in MRI enhancement kinetic curve) [10, 11]. However, this is inconclusive at the current stage due to the limited number of cases.

The specificity of our study is relatively low at 43.1%, as half of the benign lesions also demonstrated enhancement. This is, however, not far from the pooled specificity of 58% (95% CI: 38–77%) quoted by a recent meta-analysis reported by Tagliafico et al. [12] and is still within the confidence interval. We believe that the reasons for our relatively low specificity are as follows: (1) a high pretest probability might have led to overreading. This study was carried out in a diagnostic setting with the majority of patients being symptomatic. All study participants were also selected from those that had suspicious findings on mammography, tomosynthesis, or ultrasound. Retrospectively, some lesions that were classified as having mild enhancement might well have been due to background parenchymal enhancement. (2) This was a pilot study in our institution. Interpreting radiologists were faced with a steep learning curve.

The limitations of our study are as follows: (1) small sample size of a single institution. Despite having identified more than 1500 BI-RADS 4/5 lesions during the period of the study, only a handful could eventually be included in our study as the majority of the patients were unwilling to participate in this pilot study. Many patients opined that there was no direct benefit to them whilst an additional investigation with radiation and intravenous injection was required for the participation. A high proportion of patients also had contraindications to contrast injection, predominantly due to renal impairment. This may partly be contributed by the higher mean age of our patient population in our hospital's setting. This may have resulted in biased enrollment. (2) All lesions included in our study are masses and are visualized on ultrasound and hence could be biopsied under ultrasound guidance. Patients with enhancing lesions which are occult on mammogram and ultrasound would have probably needed further workup such as a MRI breast. We have excluded lesions with only calcifications as it is believed that calcification should be managed as it is regardless of its enhancement on CESM [18].

Our study added further supporting evidence with regard to the value of the high negative predictive value of CESM and provided a different perspective of its potential clinical application in reducing benign biopsy rates. We do, however, acknowledge the small sample size of our study and multicenter study, and a larger sample size would better define the role and diagnostic performance of CESM under different clinical settings. In the future, CESM may also play a bigger role in the assessment of BI-RADS 4 and 5 lesions through the quantification of the degree of enhancement with objective measurements, as well as through the study of temporal enhancement in CESM with comparison against MRI kinetic curves.

## 5. Conclusion

There is evidence that the absence of enhancement in CESM strongly favours benignity. Along with conventional imaging, it may lend sufficient confidence to the reporting radiologist to downgrade some cases, especially some of the BI-RADS 4A cases to BI-RADS 3. This can reduce up to half the number of benign biopsies, reduce the patient's physical, financial, and mental stress, and allow for better resource allocation. CESM also increases the detection rate for potentially malignant lesions, thereby changing the treatment strategies.

## Figures and Tables

**Table 1:** Histopathologic diagnosis of the 83 enhancing lesions.

Histopathology of enhancing malignant lesions (true positive)	BI-RADS 4A	BI-RADS 4B	BI-RADS 4C	BI-RADS 5	Frequency (%)
Invasive ductal carcinoma (IDC)	0	1	23	19	43 (79.6)
Invasive lobular carcinoma (ILC)	0	2	2	0	4 (7.4)
Ductal carcinoma in situ (DCIS)	0	1	1	1	3 (5.6)
Mucinous carcinoma	0	0	2	0	2 (3.7)
Mixed invasive ductal carcinoma and invasive lobular carcinoma	0	0	1	0	1 (1.9)
Invasive papillary carcinoma	0	1	0	0	1 (1.6)
**Total**	**0**	**5**	**29**	**20**	**54 (100.0)**

Histopathology of enhancing benign lesions (false positive)	BI-RADS 4A	BI-RADS 4B	BI-RADS 4C	BI-RADS 5	Frequency (%)
Fibroadenoma	4	8	1	0	13 (44.8)
Intraductal papilloma	2	4	0	0	6 (20.7)
Nodular adenosis	1	3	0	0	4 (13.8)
Benign breast tissue	0	2	0	0	2 (6.9)
Fat necrosis	0	1	0	0	1 (3.4)
Flat epithelial atypia	0	1	0	0	1 (3.4)
Benign phyllodes tumour	0	1	0	0	1 (3.4)
Ruptured cyst	1	0	0	0	1 (3.4)
**Total**	**8**	**20**	**1**	**0**	**29 (100.0)**

**Table 2:** Diagnostic performance of enhancement in contrast-enhanced spectral mammography for malignancy.

Statistic	Sensitivity	Specificity	Negative predictive value (NPV)	Positive predictive value (PPV)	Accuracy	*p* value
Value	54/54 **(100.0%)**	22/51 (43.1%)	22/22 **(100.0%)**	54/83 (65.1%)	76/105 (72.4%)	<0.001
95% confidence interval	93.4%–100.0%	29.4%–57.8%		59.5%–70.3%	62.8%–80.7%	

## Data Availability

The data are available from the corresponding author or first author on reasonable request.
